# The Significance of Paying Attention to Medical Emergencies in Medical Diagnostic Laboratories in Iran

**DOI:** 10.1155/emmi/1813732

**Published:** 2024-12-02

**Authors:** Mohammad Javad Yousefi, Mansoor Soltani, Fatemeh Mezginejad, Kosar Yousefi, Mahdi Takhviji, Mohammad Hossein Soltani

**Affiliations:** ^1^Student Research Committee, Birjand University of Medical Sciences, Birjand, Iran; ^2^School of Allied Medicine, Birjand University of Medical Sciences, Birjand, Iran; ^3^Department of Critical Care Nursing, Cardiovascular Disease Research Center, Birjand University of Medical Sciences, Birjand, Iran; ^4^Department of Hematology, School of Allied Medicine, Cellular and Molecular Research Center, Birjand University of Medical Sciences, Birjand, Iran; ^5^School of Medicine, Student Research Committee, Gonabad University of Medical Sciences, Gonabad, Iran

## Abstract

**Background:** Medical diagnostic laboratories as high-risk environments are often exposed to unpredictable situations such as patient fainting, blood pressure drops, chemical spills, and burns. These life-threatening events defined as medical emergencies and necessitate urgent actions. Hence, determining the most common medical emergencies in medical laboratories, so understanding and planning strategies to effective management seems to be crucial.

**Objective:** This study aimed to investigate medical crises in Iranian medical laboratories.

**Methods:** In this cross-sectional study, data collection was performed by a simple random sampling method through electronic and paper questionnaires filled by personnel in private and hospital laboratories in different provinces.

**Results:** The most frequent medical emergencies were patient fainting, staff needle stick, and patient's blood pressure dropping. The occurrence of medical emergencies was 24% and 76% in men and women, respectively. Out of all, treatment was administered at the scene of the accident in 37.1% of cases, and 28.1% were discharged after sampling. Moreover, 51% of the medical diagnostic laboratories had a trolley code, with injection devices and angiocaths as available tools. In 81% of the laboratories, practicing for probable medical emergencies was not possible. A significant relationship was found between the type of client (laboratory personnel or the referring person) and the type of emergency event (*p* < 0.05).

**Conclusions:** Considering the prevalence and importance of handling medical emergencies in a short time, it is necessary to design training courses for laboratory personnel and expert them to encountering with unpredictable threats in order to help affected individuals.

## 1. Introduction

Medical emergencies encompass unforeseen medical incidents necessitating immediate attention from emergency medical services (EMS) [1]. Typically, they involve critical situations wherein the patient's life is imminently threatened, such as accidents and severe trauma [2].

Medical diagnostic laboratories undertake a crucial role in disease surveillance, tracking, and occasional treatment [3, 4]. To accomplish this, they employ advanced equipment's and highly skilled personnel to analyze samples and provide precise results who worked in different departments and were involved in sampling and technical sections [4, 5].

Regrettably, medical emergencies can also occur within medical diagnostic laboratories, jeopardizing laboratory personnel and patients, so that medical emergencies may threat both visiting patients or employments engaged in requested tests [6]. Common medical emergencies encountered within medical diagnostic laboratories include convulsions and hypotension [7–9], and given the nature of these crises, any negligence or errors in treatment may have irreparable consequences for the affected individuals. However, unfortunately, precise statistical data about these incidents are currently lacking [2].

The Institute of Medicine (IOM) delineates patient safety as the ultimate objective of medical laboratory services by the prevention of harm to patients, thereby ensuring the absence of adverse events throughout the entirety of medical care processes [2]. Although, in comparison to other disciplines such as emergency and critical care, medical laboratory services are considered to possess a low level of risk; the sampling section serves as an integral department within the laboratory where personnel involve in direct communication with patients to collect patient samples [10]. However, it is worth noting that accidents may occur during the sampling process, particularly due to underlying medical conditions that some patients may experience, such as hypertension, diabetes, or even psychological disorders. Furthermore, certain individuals may experience health complications such as heart attacks or cancer, which could result in incidents such as syncope, bleeding, or dizziness. In addition, some may possess a fear of syringes and blood, potentially causing them to experience vasovagal syncope upon encountering these stimuli [11–13]. Symptoms indicative of syncopal episodes include cold, clammy skin, pallor, lightheadedness, dizziness, and nausea [14, 15]. Actually, stress and excitement potentially induce hyperventilation and subsequent respiratory alkalosis, and spasm represents another possible consequence arising from the collection of samples. Therefore, utmost care should be exercised to prevent harm to patients, and immediate assistance must be rendered in such circumstances.

On the other hand, laboratories rely on the utilization of various chemicals, including acids and identifying solutions for testing procedures. It is essential to acknowledge that certain chemicals can pose a hazard to the human body through inhalation, skin contact, or ingestion. The release of hazardous chemicals into the laboratory environment is a prevalent occurrence, potentially leading to adverse effects on the respiratory system and manifesting symptoms such as dyspnea, asthma, and respiratory system malignancies. Glass breakage or leakage from test tubes may happen, likewise, results in direct contact with the skin, thereby inducing damage and potentially giving rise to more severe pathologies [16–18].

Given that the presence of emergency medical personnel is not common in outpatient sampling departments and the risk of mentioned emergencies, laboratory staff must possess a comprehensive understanding of these situations and management. Consequently, the present study designed to investigate the various types and characteristics of medical emergencies in medical laboratories, so that it enhances awareness regarding the precautionary measures and specialized equipment employed by laboratory personnel.

## 2. Materials and Methods

In this cross-sectional study, which encompassed both descriptive and analytical approaches, a combination of electronic and paper questionnaires was used to collect data in medical laboratories in Iran from 2023 to 2024. Data collection was conducted using the simple random sampling method, and gathering information and questionnaires in this study lasts for three months (December 22, 2023, to February 20, 2024). The questionnaires covered several aspects including the following:

1. Demographic information such as age, work experience, and gender
2. The date of the medical emergency
3. Classification of the injured individual based on their role (medical personnel, service-administrative personnel, person referring to the laboratory, and companion of the patient)
4. Types of medical emergencies diagnosed in the laboratory
5. Type of care provided
6. Tools and equipment used
7. The final condition of the victim

The content validity index was utilized to ascertain the validity of the questionnaire. To accomplish the aforementioned objective, 10 specialists (five specialists in laboratory science and five specialists in emergency medicine) were given the questionnaire to evaluate its appropriateness in order to compare the questionnaire substance with the aim of the study. The Cronbach's alpha test was performed to determine the questionnaire's reliability. Ten participants were given the questionnaire at a 2-week interval, and the results showed a reliability score of 84.15%, indicating that the questionnaire was reliable.

These questionnaires were distributed to the technical personnel of the laboratory and the sampling department, randomly distributed across various provinces in Iran. The study received financial support from Birjand University of Medical Sciences (Ethical Code number: IR.BUMS.REC.1402.370).

### 2.1. Statistical Analysis

In this study, descriptive statistics were employed to illustrate the data, including mean, SD, and frequency (%). The association between drug usage and medical emergencies as well as the link between gender and medical emergencies were examined using the chi-square and fisher exact tests. The data analysis was performed by SPSS software version 16. *p* value < 0.05 was considered as a significant level.

## 3. Results

**Figure 1:**
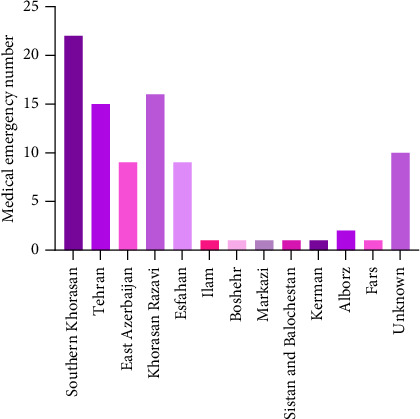
Prevalence of medical emergencies in diagnostic laboratories across Iranian provinces.

**Figure 2:**
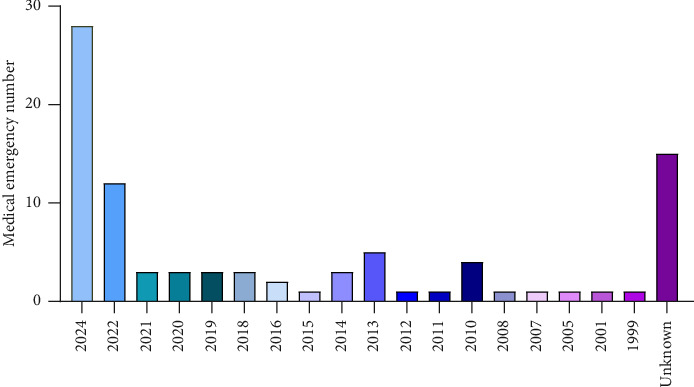
Prevalence of medical emergencies in medical diagnostic laboratories in Iran over various years.

During this study, a total of 89 paper and electronic questionnaires were completed. Data analysis revealed that the frequency of medical emergencies in government laboratories was 58.4%, while private laboratories accounted for 41.6% of such incidents. Among the provinces of the country, South Khorasan exhibited the highest prevalence of medical emergencies at 26.5%, followed by Razavi Khorasan and Tehran at 18%, and Isfahan and East Azerbaijan at 10.8%.  Figure 1 presents the distribution of medical emergencies in the country's laboratory. In the years 2023, 2022, and 1992, the highest proportions of medical deaths were recorded at 31%, 17.5%, and 6.25%, respectively (Figure 2).

**Figure 3:**
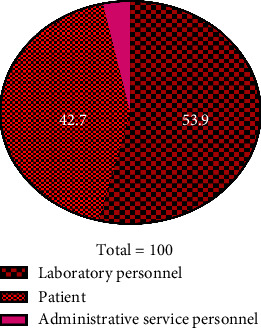
Prevalence of medical emergencies based on client type (laboratory personnel or patient or administrative service personnel).

It was observed that 75.3% of affected individuals were female, while the remaining 24.7% were male. Out of the total, 53.9% of medical emergencies were associated with laboratory personnel, 42.7% were related to healthcare professionals, and 3.4% occurred in administrative service personnel (Figure 3). The needle stick, syncope, and hypoglycemia were the most prevalent medical emergencies encountered in medical diagnosis laboratories, with frequencies of 23.6%, 20.2%, and 15.7%, respectively. Among the individuals experiencing these emergencies, 70.8% did not have any underlying diseases and out of all, 83.1% of the participants did not report using any specialized medication.

Regarding medical emergencies, 37.1% were attended at the accident site, while 28.1% were discharged after undergoing sampling procedures. In the majority of cases (62.9%), medical professionals were not called upon for assistance, whereas in 37.1% of cases, doctors or nurses summoned. Approximately half of the laboratories (51.7%) did not have trolley codes, and among those that did have (48.3%), the most commonly aid devices were injection devices, angiocaths, capsules, and oxygen cannulas.

In 55.1% of medical emergency cases, the incident report was conducted accurately and in adherence to laboratory or hospital policies. In addition, 55.1% of the lab employees had received the required training in medical emergency procedures, although 70.8% of medical diagnostic laboratories did not possess the necessary facilities to effectively carry out medical emergency management. We did not obtain any significant correlation observed between the type of medical emergency and age, and also no statistically significant relationship was identified between gender and the type of emergency (Table 1), whereas a significant relationship was found between the type of client (laboratory personnel or the referring person) and the type of emergency event (*p* < 0.05) as well as between drug consumption and the type of emergency incident (*p* < 0.05, Table 2).

## 4. Discussion

Medical emergencies require prompt medical intervention and the provision of care to the affected individuals, and medical laboratories represent a setting prone to occurrences of medical emergencies not only for patients but also for lab staff, including fainting or fluctuations in blood pressure, and it is worth noting that usually they lack required training in response to medical emergencies. Since no study has been conducted regarding the frequency of medical emergencies, as well as required equipment to encounter these situations, and specialized care in Iran's medical diagnostic laboratories thus far, this study aimed at the investigation of this issue for the first time.

The findings in this study revealed that the most prevalent medical emergency was the needle stick injuries among laboratory personnel and syncope among patients. Although there was not any study in this area for medical laboratories, we found the similar studies in other specialties including, Maryam Amir Chaghmaghi et al.'s study in 2010 performed among dentists. They stated that the most common emergency was syncope which contrasts with the findings of our study, and the potential explanation for this disparity could stem from variations in occupational roles [19] where laboratory personnel are more susceptible to needle stick injuries due to their frequent exposure in this setting, thereby increasing the likelihood of this specific medical emergency. In addition, syncope and fainting represent the second most prevalent medical emergency encountered in the sampling process. Such reactions are often triggered by the fear of sampling needles and syringes or by negative past experiences associated with the sampling procedure.

Based on our findings, it was identified that the South Khorasan province had the highest incidence of medical emergencies, while the provinces of Ilam, Fars, Bushehr, Makasi, and Kerman had the lowest incidence, respectively. However, we assume that this discrepancy may be attributed to variations in the number of questionnaires obtained from each province, and it is plausible that different outcomes could be obtained with a larger sample size in future studies.

Our findings demonstrated that there is no significant relationship between the incidence of medical emergencies and the age or gender of the accident victims. These results differ slightly from those of Mohammad Rumanian and Tawfiq's study in dentistry, which reported a significant relationship between the incidence of medical emergencies and the age and gender of the victims [20]. This discrepancy may be attributed to the differences in laboratory practices and tools used between dentistry and medical diagnostics. However, it was observed that the occurrence of medical emergencies was more prevalent among women compared to men. This phenomenon may be attributed to women heightened emotional and physical sensitivity, as well as their fear of stimuli such as blood, needles, and other related factors.

Regarding the lab staff training condition, Babai Hatke Loui and Shiva's study in 2014 showed that 53% of individuals had successfully completed the medical emergency courses which are in accordance with our results indicating 55.1% of participants had received adequate training in dealing with medical emergencies [21]. For instances, it is crucial for medical staff to know to encounter with a vasovagal syncope situation, so that individuals should assume a supine position with their heads lowered along with using a cold compress on the forehead and the posterior part of the neck till the individual's consciousness is restored.

Moreover, in our study, 48.7% of the laboratories had the trolley code. This was similar to the results of Mehdi Babaei Hatke Loui et al.'s study, where 39.3% of dentists were found to possess emergency kits [21]. So considering the prevalence and significance of medical emergencies, it is essential for all laboratories to equip themselves with trolley codes and emergency kits. Nevertheless, laboratory personnel ought to receive required training that encompasses diverse scenarios and equips them to manage such incidents effectively.

Given the more prevalence of medical emergencies among individuals with underlying illnesses or those taking specific medications, this issue may show a significant risk factor, although caution should be used in interpreting these results of correlation evaluation. Actually, our study only identifies correlations, not causality and in order to confirm causality relationship more specific studies demanded. Nevertheless, it is imperative that personnel in medical diagnostic laboratories are equipped to handle these cases and adhere to the necessary safety protocols. In addition, laboratory personnel can implement a protocol within the sampling department to collect the patient's history and demographic information. This proactive approach aims to enhance cautionary measures, specifically for individuals who exhibit a heightened susceptibility to medical emergencies. Necessary tools and equipment for managing injured individuals should be readily accessible and comprehensive, ensuring prompt and precise intervention for such individuals.

Consequently, based on the findings derived from this study and despite some limitations, heightened focus should be directed towards training and familiarizing personnel within medical diagnostic laboratories with the necessary knowledge and skills to correctly manage and assist accident victims. Furthermore, it is essential that all laboratories should be equipped with the necessary trolley codes and emergency equipment, and emergency medical personnel can be designated as part of the staff within medical diagnostic laboratories. In addition, it is plausible to impart the required skills and provide training to one of the laboratory personnel, enabling them to effectively handle medical emergencies, thus ensuring their usefulness in the case of accidents or unforeseen medical situations.

It is noteworthy that this study represents the first investigation into the prevalence of medical emergencies specifically within medical diagnostic laboratories in Iran, and to date, no research has been conducted in this specific domain. So this study has the potential to serve as a precursor for subsequent studies, encompassing a larger sample size and a wider range of data collected from laboratories in various regions across the country.

## 5. Conclusion

Given the results of our study and the significance of medical emergencies in medical laboratories, it becomes imperative to implement measures to organize training sessions for laboratory personnel and equip them with the necessary skills to effectively and promptly manage potential risks. So in such situations, healthcare professionals can provide timely assistance to patients and affected individuals.

## Figures and Tables

**Table 1:** The incidence rate of gender-based medical emergencies.

Medical emergency	Hypotension (%)	Syncope (%)	Needle stick (%)	Angina (%)	Hypoglycemia (%)	Pant (%)	Splashing (%)	Others (%)	*p* value
Sex									
Male	13.6	13.6	22.7	13.6	18.2	4.5	9.1	4.5	*p* > 0.205
Female	16.4	26.9	23.9	0	14.9	4.5	7.5	6	

**Table 2:** The incidence rate of medical emergencies in relation to drug usage.

Medical emergency	Hypotension (%)	Syncope (%)	Needle stick (%)	Angina (%)	Hypoglycemia (%)	Pant (%)	Splashing (%)	Others (%)	*p* value
Medication usage	26.7	6.7	20	20	6.7	6.7	6.7	6.7	*p* < 0.018
Not taking medicine	15.7	23.6	23.6	3.4	15.7	4.5	7.9	5.6	

## Data Availability

The data that support the findings of this study are available from the corresponding author on request. The data are not publicly available due to privacy or ethical restrictions.
